# Temporal Changes of Spinal Transcriptomic Profiles in Mice With Spinal Nerve Ligation

**DOI:** 10.3389/fnins.2019.01357

**Published:** 2019-12-17

**Authors:** Hong Yu, Piao Zhang, Ye-Ru Chen, Yong-Jie Wang, Xian-Yi Lin, Xiang-Yao Li, Gang Chen

**Affiliations:** ^1^Department of General Surgery, Sir Run Run Shaw Hospital, School of Medicine, Zhejiang University, Hangzhou, China; ^2^Department of Anesthesiology, Sir Run Run Shaw Hospital, School of Medicine, Zhejiang University, Hangzhou, China; ^3^Institute of Neuroscience and Collaborative Innovation Center for Brain Science, School of Medicine, Zhejiang University, Hangzhou, China

**Keywords:** neuropathic pain, spinal nerve ligation, RNA-Seq, integrated differential expression and pathway analysis, transcriptomic profiles

## Abstract

Neuropathic pain (NP) is an intractable disease accompanying with allodynia, hyperalgesia and spontaneous pain. Accumulating evidence suggested that large volume of neurotransmitters, genes, and signaling pathways were implicated with the initiation and development of NP, while the underlying mechanism still remained poorly understood. Therefore, it was extremely important to further elucidate the potential regulatory networks for developing appropriate treatment options. Here, the RNA-Seq high-throughput sequencing was employed to determine the genes expression change in mice undergoing spinal nerve ligation (SNL). Meanwhile, the differentially expressed genes (DEGs) were analyzed by using integrated Differential Expression and Pathway analysis (iDEP) tools and String database. Then, quantitative real-time PCR (qRT-PCR) was employed to detect the expression of hub gens. The results showed that the DEGs mainly comprised 1712 upregulated and 1515 downregulated genes at 7 days, and consisted of 243 upregulated and 357 downregulated genes at 28 days after surgery, respectively. Additionally, 133 genes and two pathways including retrograde endocannabinoid signaling and cardiac muscle contraction collectively participated in biological reactions of 7th and 28th day after operation. Moreover, the results showed that the mRNA and protein expression of Ccl5, Cacna2d1, Cacna2d2, Cacnb2, Gabrb3, GluA1, and GluA2 were significantly upregulated in SNL-7/28d group than that of in Sham-7/28d group (SNL-7d vs. Sham-7d; SNL-28d vs. Sham-28d; *P* < 0.05). And the level of Glra2, Glra4, Glra3, Grik1, Grik2, NR1, NR2A, and NR2B was obviously increased in SNL-7d group compared to Sham-7d group (*P* < 0.05), but which was no statistical difference between SNL-28d group and Sham-28d group. Therefore, these results provided new perspectives and strategies for deeply illuminating the underlying mechanism, and identifying the key elements for treating NP.

## Introduction

Neuropathic pain (NP) is one of the most intractable diseases threatening human health, which mainly characterized by chronic pain accompanying with allodynia, spontaneous pain and hyperalgesia (Murnion, 2018; Nent et al., 2019). NP is not one disease entity but a cluster of symptoms derived from a primary lesion, dysfunction or transitory perturbation comprising of peripheral and central nervous systems (Murnion, 2018). Currently, epidemiological investigations showed that the prevalence of NP was approximately 6.9–10% worldwide, and the puzzle became increasingly prominent with social development (Gilron et al., 2015). Through dozens of years of unremitting efforts, the advances in the field of medicine provided adequate treatment strategies including opioid analgesics, non-steroidal anti-inflammatory drugs, and traditional drugs assisted by minimally invasive pain therapy, ultra-laser radiation and individualized psychotherapy to effectively ameliorate the NP symptoms (Inoue, 2006; Wright and Rizzolo, 2017). Unfortunately, all these therapeutic methods could not completely cure NP. Recent researches showed that nervous system injury, diabetes, virus infection, and neurotoxicity of drugs/radiotherapy were the principal causes resulting in NP (Inoue, 2006). Additionally, emerging evidence documented that an imbalance of the excitatory and inhibitory neurons (Li et al., 2019), or sensitization of neurons directly enhanced neuronal excitability and thereby contributed to nerve ectopic discharge and abnormal nociceptive signal transmission (Willis, 2001; Moutal et al., 2019; Nent et al., 2019). Whereas, the underlying mechanism was still complex and difficult to illuminate thoroughly stemmed from numerous neurotransmitters, ion channels, receptors, and signaling pathways were involved in the occurrence and development of NP (Gonzalez-Ramirez et al., 2017). Therefore, it is extremely essential to further elucidate the molecular mechanisms for developing appropriate treatment schedules.

Next-generation sequencing (NGS) is a rapidly developing domain that tremendously propelled the research space and infiltrated clinical applications with enormous influence (Kumar and Cowley, 2019). Meanwhile, the emergence of NGS has revolutionarily driven the development of life sciences and medicine by accelerating the research of various genomics and providing new paradigm for the diagnosis and treatment of complex diseases (Baudhuin, 2012; Buermans and den Dunnen, 2014). As informatics tools and analysis pipelines, NGS has unique superiority in processing massively parallel sequencing reactions simultaneously with a rapid and cost-effective manner (Le Gallo et al., 2017). Additionally, NGS could provide numerous actionable information for clinicians and public health specialists, and dramatically improve accurate diagnosis for diseases (Besser et al., 2018). Moreover, large-scale volumes of multi-faceted data would be meaningfully assembled, mined, retrospectively analyzed and therefore provide valuable outcomes for disease therapy (Giannopoulou et al., 2019). Collectively, comprehensively analyzing and rationally interpreting data obtained from NGS facilitated to screen target genes and key pathways for treating diseases. Moreover, we systematically reviewed the previous researches concerning the pain areas. Interestingly, the results revealed that RNA-Seq technology was widely employed to investigate the underlying mechanism related to pain (Supplementary Table S1). However, no relative experiments were performed to dynamically detect the molecular expression and analyze potential mechanism. Therefore, we speculated that these genes may play different roles in different time points after surgery. The illustration of space-time specialty about differentially expressed genes (DEGs) would conduce to further identify key therapy targets for NP. However, there were no related reports concerning the dynamic role of DEGs. Consequently, to investigate the potential molecular mechanism, the specimens harvested from mice at different time points following spinal nerve ligation (SNL) were sequenced by using RNA-Seq technology. And the DEGs were analyzed via network online tools including integrated Differential Expression and Pathway analysis (iDEP) tools and String database. Then, the relationship networks of Gene Ontology and KEGG Pathway Analysis of DEGs were portrayed to exhibit the conceivable patterns regarding to NP evolution with time. These results provided a fresh perspective for thoroughly comprehending the underlying mechanism and developing appropriate therapy methods about NP.

## Materials and Methods

### Animals and Ethics Statement

Adult male C57/BL6 mice (8 weeks old, 25–30 g) were purchased from the Experimental Animal Center of Zhejiang University. All experimental procedures were approved by the Animal Care and Use Committee of Zhejiang University, and in accordance with guidelines for laboratory animal care and safety from NIH. All animals were housed individually with unrestricted access to chow and water in standard environmental conditions (temperature: 22–25°C, humidity: 45–50%, and 12 h light/dark cycle).

### Establishment of Spinal Nerve Ligation Model

The spinal nerve ligation (SNL) model was established as previously described (Kim and Chung, 1992). Briefly, animals were anesthetized intraperitoneally with 2% pentobarbital sodium (40 mg/kg). The back skin was incised longitudinally, and the transverse processes of the sixth lumbar vertebra was excised to expose the spinal nerves. Subsequently, the left L5 and L6 spinal nerves were carefully isolated and ligated tightly using 6-0 silk. After checking hemostasis, the adjacent fascia and tissue were ligated with a 4.0 nylon thread and the skin was closed with sutures. Sham group underwent the same procedure, while the exposed spinal nerves (L5 and L6) were not ligated. All animals were allowed to recover in an individual cage for 3 days after surgery.

### Tissue Harvest

Animals were anesthetized with 2% pentobarbital sodium (40 mg/kg, i.p.) at 7 and 28 days after surgery. Then, an incision was made in the right atrium and transcardiac perfusion with heparinized 0.9% saline followed by 4% formaldehyde was performed. The spinal cord (L4–L6) tissues were obtained, and the samples were post-fixed for 4 h at 4°C. Meanwhile, the tissues were kept in 30% sucrose in 0.1 M phosphate buffers (pH 7.4, 4°C) for 3 days. Then, the specimens were stored in a −80°C freezer.

### RNA Extraction

Total RNA was isolated and extracted from SNL-7d group and SNL-28d group through RNAiso Plus Reagent (TaKaRa, Japan), then purified using RNasey Mini Kit (QIAGEN) according to the manufacturer’s protocol. The RNA concentration was detected through NanoDrop, and electrophoresis was used to confirm the integrity as quality control. The cDNA synthesis and antisense RNA (aRNA) amplification were performed by using Amino Allyl MessageAmp II aRNA Amplification Kit (Ambion, United States). The total RNA was stored at −80°C for later experiment. Three biological replicates were used in this study.

### RNA-Seq Data Acquisition

A total of 1.5 μg RNA was used as the input material. The clustering of the index-coded samples was employed using a TruSeq PE Cluster Kit v3-cBot-HS (Illumina) based on the manufacturer’s instructions. The library were sequenced on an Illumina HiSeq platform by Novogene (Beijing, China), and paired-end reads were generated following cluster generation. Therefore, raw reads in the fastq format were processed via in-house Perl scripts. Then, low-quality data were discarded from raw reads using Trim Galore^1^. The GC-content and sequence duplication level of the clean reads were calculated, and the clean reads were assembled with Trinity software using the default parameters^2^. Then, the RNA-Seq data files were deposited in the NCBI Sequence Read Archive (SRA) database (SRA accession: PRJNA589837).

### Data Analysis Using Integrated Differential Expression and Pathway Analysis (iDEP) Tools

The DEGs obtained from RNA-Seq-Based expression profiling were analyzed by using iDEP (integrated Differential Expression and Pathway analysis) online tools^3^. iDEP seamlessly connects 63 R/Bioconductor packages, 2 web services, and comprehensive annotation and pathway databases for 220 plant and animal species. For details on the process, refer to the manual that externally published papers (Ge et al., 2018). Briefly, expression matrix of DEGs was filtered and converted to Ensemble gene IDs, and the pre-processed data was used for exploratory data analysis (EDA) including *k*-means clustering and hierarchical clustering. The pairwise comparison (SNL-7d group vs. Sham-7d group; SNL-28d vs. Sham-28d group) was performed using the DESeq2 package with a threshold of false discovery rate (FDR) < 0.5 and fold-change > 1.5. Moreover, a hierarchical clustering tree and network of enriched GO terms were constructed to visualize the potential relationship. Gene Set Enrichment Analysis (GSEA) is a computational method that determines whether an *a priori* defined set of genes shows statistically significant between two biological states. Therefore, GSEA method was performed to investigate the related signal pathways activated by surgical operation. Moreover, identify co-expression networks and sub-modules were constructed by using WGCNA, and the enriched pathways in selected module were exhibited, respectively.

### Gene Ontology and KEGG Pathway Analysis of DEGs

Gene ontology (GO) analysis and Kyoto Encyclopedia of Genes and Genomes (KEGG) pathway were applied to analyze the differentially expressed genes (DGEs) between SNL-7d group and SNL-28d group using String online tools^4^. GO analysis was utilized to annotate genes and gene products consisting of molecular function (MF), biological process (BP), and cellular component (CC) (Gene Ontology Consortium, 2006). KEGG is a knowledge base for systematic analysis of gene functions comprising a series of genome and enzymatic approaches and genomic information with higher order functional information (Kanehisa and Goto, 2000), which is used for systematic analysis of gene function and related high-level genome functional information of DGEs.

### Integration of Protein–Protein Interaction (PPI) Network Analysis

STRING version 10.0 covers 9, 643, 763 proteins obtained from 2031 organisms (Szklarczyk et al., 2015). The String database^4^ is utilized to assess and predict the protein-protein interactions comprising direct (physical) and indirect (functional) associations. To assess the interactional relationships and build a PPI network between SNL-7d group and SNL-28d group, String tool was employed and PPI network was established according to the function and pathway enrichment analysis.

### Quantitative Real-Time PCR (qRT-PCR)

The animals were sacrificed and the spinal cord tissues (L4-L6: 10-mm-long around the injury site) were harvested at 7 and 28 days after operation injury. The mRNA of hub genes (including Cacna1i, CCL5, Glra2, Glra4, Glra3, Cacna2d1, Cacna2d2, Cacnb2, Ccl21a, Gabrb3, GluA1, GluA2, Grik1, Grik2, Grik3, NR1, NR2A, NR2A-1, NR2B) predicted by bioinformatics methods was assessed by using qRT-PCR. Briefly, the total RNA of the spinal cord samples was isolated utilizing TRIzol reagent (superfecTRITM) according to the manufacturer’s protocol (Invitrogen), and reverse transcribed to cDNA with the Revert Aid TM First Strand cDNA Synthesis kit (Thermo Scientific). The forward and reverse primer sequences used in this study as showed in Table 1. PCR amplification was carried out as follows: (1) Initial denaturation (1 cycle, 95°C for 3 min), (2) Denaturation (40 cycles, 95°C for 15 s), (3) Amplification (40 cycles, 53°C for 30 s, and 60°C for 40 s). The PCR products were verified by 1% agarose gel electrophoresis, visualized by Goldview (WOLSEN) staining. The gels were analyzed by Alpha Innotech (Bio-Rad), and optical density (OD) was measured for statistical analysis.

**TABLE 1:** Information for primer sequences.

**Gene**	**Upstream**	**Downstream**	**Length**
Cacna1i	5′-GATCAGACTGTGTGGCCGCCAACTA-3′	5′-CCTTGGAGGCCAAGACAAAGAGGGA-3′	102 bp
Cacna2d1	5′-GGAGGACCTATTCAGTGGATGGCTTG-3′	5′-GTCCATTGAACTTGCTTCGCTTTGT-3′	154 bp
Cacna2d2 Cacnb2 Cacnb4 Gabra3 Gabra4 Gabrg3	5′-TCATCCAGTTTGGGTGACATAGTGC-3′ 5′-TCATCCAGTTTGGGTGACATAGTGC-3′ 5′-GAACATTCCGAGCAACTCCCACAAC-3′ 5′-GGGACCCTCCTCTATACAATGAGGT-3′ 5′-ACCTCAGACGGAAGATGGGCTACTT-3′ 5′-ATCATGGCGGCTCTATCAGTTTGAC-3′	5′-TCGGCTGCCTTGTTCGGGA-3′ 5′-CCACATCATAAGGAGGAGTGTGCTC-3′ 5′-TCAGTGATGGCCCCACTAACACCAC-3′ 5′-CCAAGCAAGTCATACTGATTCAGGC-3′ 5′-AGCACTGATGCTTAGGGTGGTCATC-3′ 5′-AATGACACCCAGGACAGAACCACAG-3′	142 bp 220 bp 120 bp 212 bp 167 bp 183 bp
Gabrq	5′- CATATGCGGGATAAACTCCCCCATA-3′	5′- TTGCTACCACCACTTCTTGGTAGCG-3′	211bp
Cc15 Gabrb1 Gabrb2 Gabrb3 Glra2 Glra3	5′-GACACCACTCCCTGCTGCTTTG-3′ 5′-AGCGATTGACATCTATCTCATGGGC-3′ 5′-ACCTCCGGGAAACTCTCCCTAAAAT-3′ 5′-TACCCACTGGATGAGCAAAACTGCA-3′ 5′-CTTCAGTGGCTATGGGATGGGTCAC-3′ 5′-AAGGACTTGCGGTACTGCACTAAAC-3′	5′-TCTTGAACCCACTTCTTCTCTGGGT-3′ 5′-CTTGTTTGCTCGCTCCCTTTTTCTG-3′ 5′-CTTTCTCAGCTGCTTTCTTTTGGCG-3′ 5′-ATAGGCACCTGTGGCGAAGACAACA-3′ 5′-GATAGCATCTGCATCTTTGGGGGGT-3′ 5′-GACCCAGGATAGAATAACGATCAGG-3′	164 bp 122 bp 162 bp 189 bp 109 bp 144 bp
Glra4 Grik1 Grik2 Grik3	5′-GTGTAGGCCTTGGCATCACCACAGT-3′ 5′-CGCGGCACAGTCCTTATCC-3′ 5′-CAGCGTCGGCTCAAACATAAG-3′ 5′-AGGTCCTAATGTCACTGACTCTC-3′	5′-GGCAGCAAATACAAAGAGCAGGCAT-3′ 5′-CACTTGAGGGGAGGTCTGA-3′ 5′-GGTTTCTTTACCTGGCAACCTT-3′ 5′-TGCCATAAAGGGTCCTATCAGAC-3′	137 bp 102 bp 105 bp 107 bp
β-actin	5′-GAAGATCAAGATCATTGCTCCT-3′	5′-TACTCCTGCTTGCTGATCCA-3′	227 bp

### Western Blot Assay

The spinal cord tissues (L4–L5) were homogenized in RIPA buffer (Beyotime, P0013B) with 1 × protease inhibitor cocktail (Beyotime, P1010). The supernatant was collected by centrifugation at 12,000 × *g* for 10 min, and the protein concentration was determined by a bicinchoninic acid protein assay kit (Beyotime, P0012S). An aliquot of 50 μg protein from each sample was separated by using SDS-PAGE and transferred to a PVDF membrane, which was blocked with 5% nonfat milk in TBST (pH 7.4). The membranes were incubated with primary antibodies (Table 2) at 4°C overnight. Secondary antibodies conjugated with HRP against rabbit IgG were performed for 2 h at room temperature and blots were exposed to a chemiluminescent detection system using the SuperSignal West Pico Substrate (34077, Pierce) and exposed to film. Digital images were quantified using densitometric measurements by Quantity-One software (Bio-Rad).

**TABLE 2:** Primary antibody.

**Primary antibody**	**Company**	**Source**	**Concentration**	**Catalog numbers**
CACN2D1	ABclonal	Rabbit	1:200	A15260
CACN2D2	ABclonal	Rabbit	1:200	A10267
CCL5	ABclonal	Rabbit	1:500	A14192
GABRB3	ABclonal	Rabbit	1:500	A10015
CACNB2	ABclonal	Rabbit	1:500	A16037
GluA1	ABclonal	Rabbit	1:500	A0111
GluA2	davagbio	Rabbit	1:1000	db558
Actin	ABclonal	Rabbit	1:10000	AC026
Anti-rabbit IgG (H + L)	Jackson ImmunoResearch	Goat	1:10000	111-035-003

### Statistical Analysis

All data were represented as mean ± standard deviation (SD). The results were analyzed by one-way analysis of variance (ANOVA) and Tukey’s *post hoc* test by using SPSS 17.0 software. *P* < 0.05 was considered as statistical significance.

## Results

### Pre-processing and Exploratory Data Analysis

**FIGURE 1:**
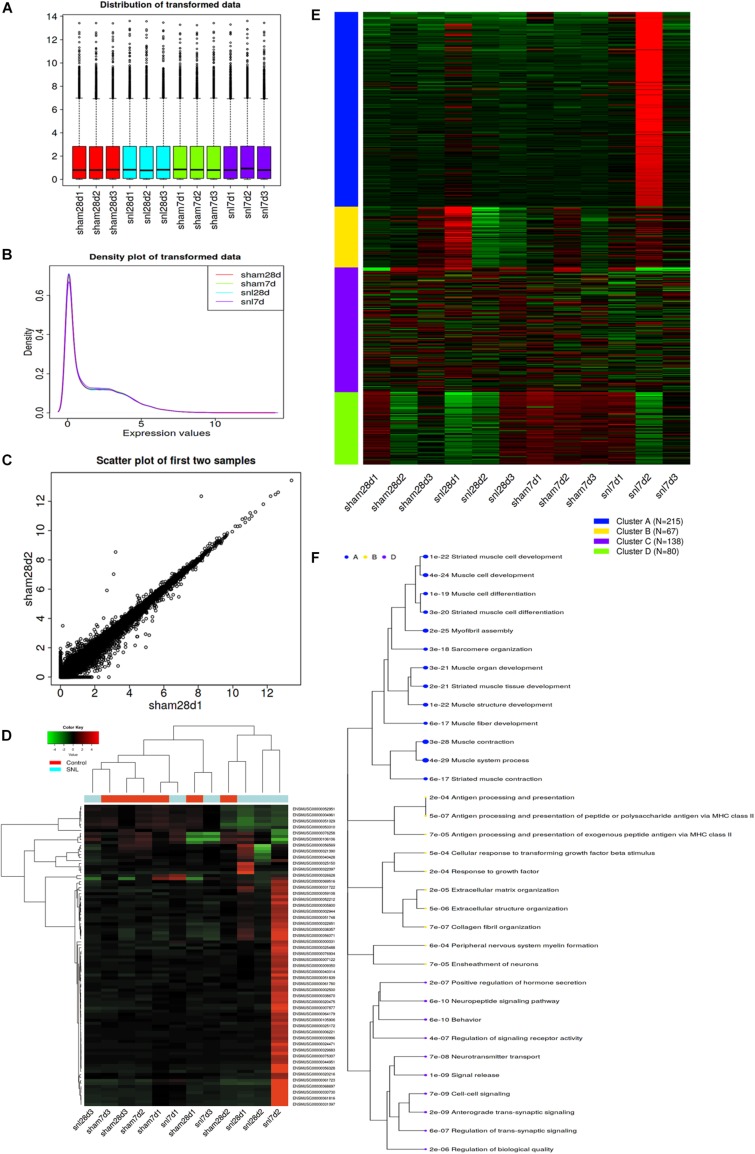
Exploratory data analysis for read-counts data. **(A)** Distribution of transformed data by using a density plot; **(B)** Boxplot of transformed data; **(C)** Scatter plot of the first two samples; **(D)** Hierarchical clustering of top 1000 genes, and the color scale represents the relative expression of genes in certain slide: green indicates low relative expression levels; red indicates high relative expression levels; black indicates zero (no change); **(E)** *k*-means clustering; **(F)** Enrichment analysis for Go terms. Sizes of dot correspond to adjusted *P*-values.

iDEP correctly recognized samples according to the number of matched genes IDs after uploading the read count data of mice with SNL. The gene ID was converted and filtered. Figures 1A,B showed the distribution of the transformed data, and Figure 1C indicated that the variation among replicates was small. Then, all genes were ranked based on the standard deviation and the hierarchical clustering was drawn across all specimens by using the top 500 genes (Supplementary Data Sheet S1). The result showed that surgical operation elicited a significant change (Top100 genes) among the genes expression (Supplementary Data Sheet S2 and Figure 1D).

### Data Analysis by Using *k*-means Clustering

The top 500 genes were divided into four groups using *k*-means clustering according to the within-group sum of squares plot. Figures 1E,F separately shows the four gene clusters and the enriched GO terms, and the data details are attached to Supplementary Data Sheet S3, S4. The result showed that Genes in cluster A related to synaptic signaling and neuropeptide signaling pathway, which may respond to nociceptive stimulus (Figures 1E,F).

### Differentially Expressed Genes (DEGs)

**FIGURE 2:**
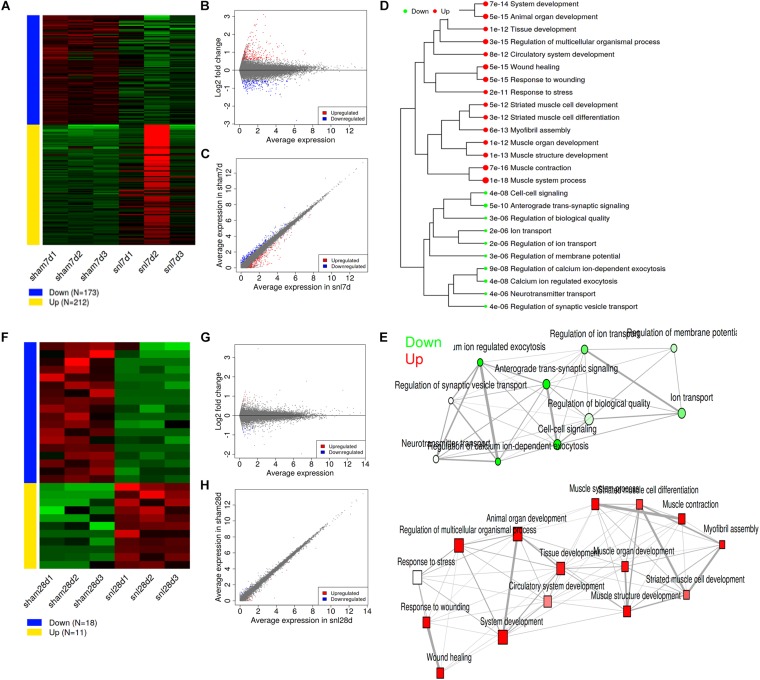
Analysis for differentially expressed genes. **(A)** Expression patterns of selected DEGs; **(B)** MA plot for DEGs; **(C)** Scatter plot for DEGs; **(D)** Hierarchical clustering tree for DEGs; **(E)** Enrichment network for DEGs; (**A–E** showed SNL-7d group vs. Sham-7d group); **(F)** Expression patterns of selected DEGs; **(G)** MA plot for DEGs; **(H)** Scatter plot for DEGs; (**F–H** showed SNL-28d group vs. Sham-28d group).

With the DESeq2 package, we identified 212 upregulated and 173 downregulated genes in SNL-7d group compared to that of in Sham-7d group (Figure 2A and Supplementary Data Sheet S5), and 11 upregulated and 18 downregulated genes in SNL-28d group compared with Sham-28d group (Figure 2F and Supplementary Data Sheet S6) by using a threshold of false discovery rate (FDR) < 0.5 and fold-change > 1.5. The MA plot (Figures 2B,G), and scatter plot (Figures 2C,H) suggest that surgical procedure results in a transcriptomic response. Moreover, the distance was measured among the terms by the percentage of overlapped genes, then a hierarchical clustering tree (Figure 2D) and network (Figure 2E) were constructed to visualize the relationship among enriched GO terms (SNL-7d group vs. Sham-7d group).

### Analysis for Pathways by GSEA Method

**FIGURE 3:**
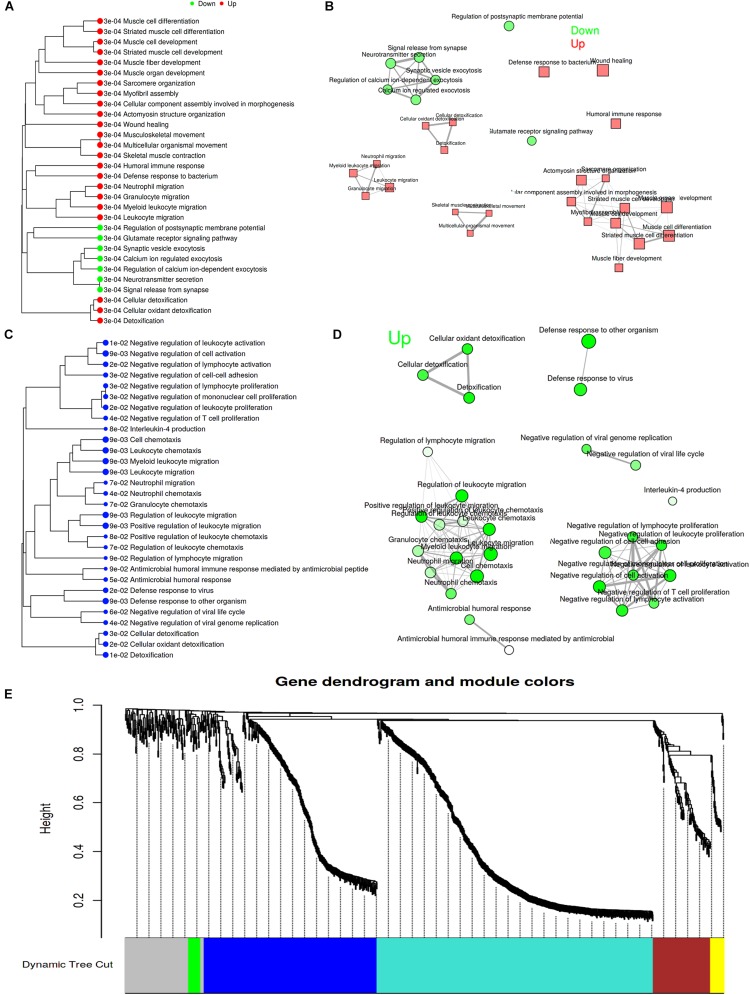
Analysis for pathways by GSEA method. **(A,C)** Pathway tree (Sizes of dot correspond to adjusted *P*-values); **(B,D)** Pathway network (Color of node correspond to adjuested *P*-values) (**A,B** showed SNL-7d group vs. Sham-7d group; **C,D** showed SNL-28d group vs. Sham-28d group); **(E)** Module partition trees of datasets, and modules are indicated by different colors.

Gene Set Enrichment Analysis (GSEA) is a computational method that determines whether an *a priori* defined set of genes shows statistically significant between two biological states. Therefore, GSEA method was performed to investigate whether surgical operation could obviously activate related signal pathways in this study. GSEA ignores gene sets that contain fewer than 15 genes or more than 500 genes, and FDR < 0.1. Figures 3A,B showed the top 30 pathway tree and network, and the detailed information was attached to Supplementary Data Sheet S7 (SNL-7d group vs. Sham-7d group). Similarly, Figures 3C,D showed the top 30 pathway tree and network, and the detailed information was attached to Supplementary Data Sheet S8 (SNL-28d group vs. Sham-28d group). Additionally, identify co-expression networks and sub-modules were constructed by using WGCNA. The results showed that a total of five modules (turquoise, blue, brown, yellow, and green) were obtained (Figure 3E). And the enriched pathways among all genes in selected module were exhibited in Supplementary Data Sheet S9.

### GO Function and PPI Network Analysis of the DEGs at 7th Day After Surgery

**FIGURE 4:**
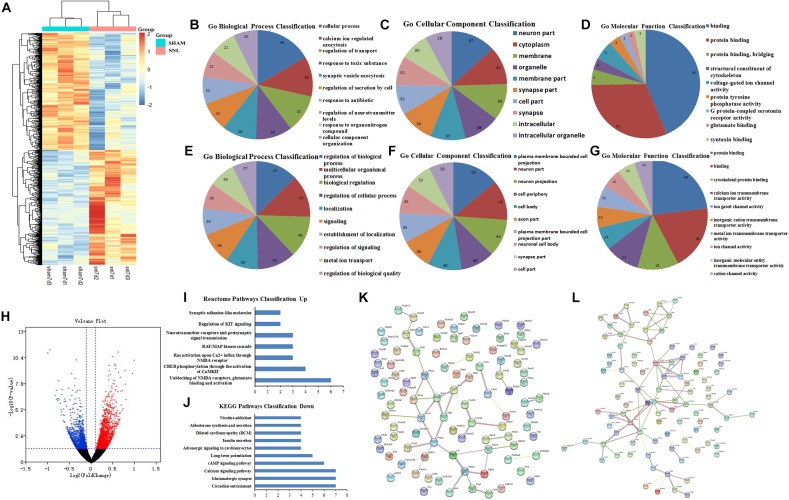
GO function and PPI network analysis of the DEGs at 7th day after surgery. **(A)** Heat map analysis of DEGs between Sham-7d group and SNL-7d group (The color scale shows the relative gene expression in certain slide: blue indicates low relative expression levels; red indicates high relative expression levels; yellow indicates no change); **(B–G)** GO function analysis of the upregulated/downregulated DEGs including biological process, cellular component and molecular function; **(H)** volcano plot of DEGs in metastasis samples compared to primary samples (Red indicates the gene expression was upregulated in SNL-7d group compared with Sham-7d group (*P* < 0.01); Blue indicates the gene expression was downregulated in SNL-7d group compared with Sham-7d group (*P* < 0.01); Black indicates *P* > 0.01); **(I,J)** Enriched pathway analysis of DEGs; **(K,L)** protein–protein interaction networks of DEGs.

The DEGs at 7th day after surgery were showed in the heat map and volcano plot, which comprised 1712 upregulated and 1515 downregulated genes (Figures 4A,H and Supplementary Data Sheet S10). Then, the GO function analysis of top 200 DEGs (100 upregulated and 100 downregulated genes) was performed by using String database. The GO as a dynamic controlled vocabulary is used to describe the role of gene with three categories information including biological process (BP), cellular component (CC), and molecular function (MF). In this study, the genes were analyzed in relevant biological process by using gene annotations. GO term enrichment analysis indicated that the upregulated DEGs were involved in 57 categories, and the top 10 BP was presented in Figure 4B; the CC consists of 52 categories, and the top 10 CC was showed in Figure 4C; and the MF includes 9 categories, which was showed in Figure 4D. Similarly, the GO function analysis of downregulated DEGs were conducted and the results were presented in Figures 4E–G including BP (Top10 of 174), CC (Top10 of 96), and MF (Top10 of 55). Meanwhile, the enriched pathways of upregulated and downregulated DEGs were showed in Figures 4I,J, respectively. Moreover, the PPI network analysis was constructed for upregulated and downregulated DEGs, and the results were separately showed in Figures 4K,L.

### GO Function and PPI Network Analysis of the DEGs at 28th Day After Surgery

**FIGURE 5:**
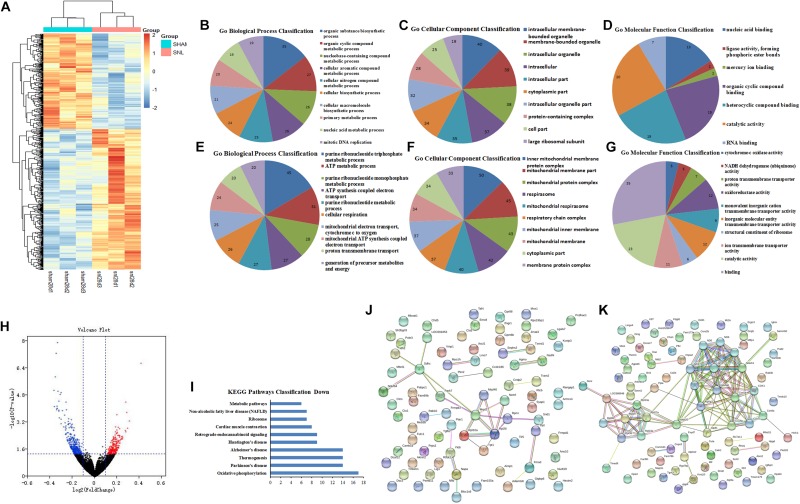
GO function and PPI network analysis of the DEGs at 28th day after surgery. **(A)** Heat map analysis of DEGs between Sham-7d group and SNL-7d group (The color scale shows the relative gene expression in certain slide: blue indicates low relative expression levels; red indicates high relative expression levels; yellow indicates no change); **(B–G)** GO function analysis of the upregulated/downregulated DEGs including biological process, cellular component and molecular function; **(H)** volcano plot of DEGs in metastasis samples compared to primary samples (Red indicates the gene expression was upregulated in SNL-7d group compared with Sham-7d group (*P* < 0.01); Blue indicates the gene expression was downregulated in SNL-7d group compared with Sham-7d group (*P* < 0.01); Black indicates *P* > 0.01); **(I)** Enriched pathway analysis of DEGs; **(J,K)** protein–protein interaction networks of DEGs.

The heat map and volcano plot of DEGs were separately showed in Figures 5A,H (Supplementary Data Sheet S11) after 28 days of operation, which comprised 243 upregulated and 357 downregulated genes. Then, String database was used to analyze GO function of top 200 DEGs (100 upregulated and 100 downregulated genes). GO function analysis of upregulated DEGs indicated that BP, CC, and MF mainly comprised of 28, 23, and 7 categories, and the top10 was showed in Figures 5B–D, respectively. Meanwhile, the results of GO function analysis of downregulated DEGs including top10 of BP (67), CC (39), and MF (10) were showed in Figures 5E–G. KEGG pathway analysis suggested that 10 pathways were enriched in the downregulated DEGs (Figure 5I). Moreover, the PPI network relationship of upregulated and downregulated DEGs was constructed and showed in Figures 5J,K.

### GO Function and PPI Network Analysis for the Conjunct DEGs at 7th and 28th Day After Surgery

**FIGURE 6:**
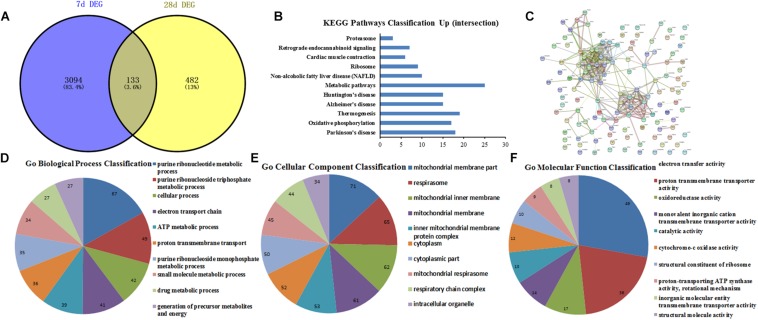
GO function and PPI network analysis of the conjunct DEGs at 7th and 28th day after surgery. **(A)** The intersection genes between 7 and 28 days DEGs; **(B)** KEGG Pathways Classification of intersection genes; **(C)** protein–protein interaction networks of intersection genes; **(D–F)** GO function analysis of the intersection genes including biological process, cellular component and molecular function.

The conjunct DEGs at 7th and 28th day after surgery were screened using the Venny 2.1 tools^5^. The results showed that 133 genes jointly participated in biological reactions of 7th and 28th day after surgery (Figure 6A). Thereafter, the conjunct DEGs were analyzed by String database. The results of GO function analysis of conjunct DEGs consisting of BP, CC, and MF included 85, 49, and 22 categories, and the top10 was showed in Figures 6D–F, respectively. Additionally, the results of KEGG pathway analysis and PPI network relationship of conjunct DEGs were showed in Figures 6B,C separately.

### GO Function and PPI Network Analysis for the Conjunct DEGs Involved in the Same Pathways at 7th and 28th Day After Surgery

**FIGURE 7:**
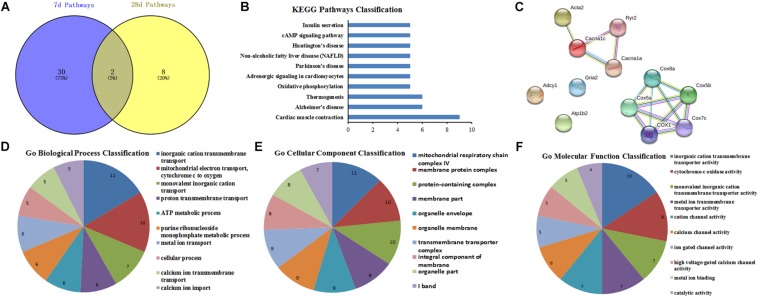
GO function and PPI network analysis of intersection genes of the conjunct pathways at 7th and 28th day after surgery. **(A)** The intersection pathways between 7 days group and 28 days group; **(B)** KEGG Pathways Classification of intersection genes of the conjunct pathways; **(C)** protein–protein interaction networks of intersection genes of the conjunct pathways; **(D–F)** GO function analysis of the intersection genes of the conjunct pathways including biological process, cellular component, and molecular function.

The conjunct DEGs of the same signaling pathways at 7th and 28th day after surgery were screened through the Venny 2.1 tools. The results showed that two pathways including retrograde endocannabinoid signaling and cardiac muscle contraction collectively responded to the surgical operation (Figure 7A). Then, the related genes involved in the same pathways were analyzed by String database. The top10 BP, CC, and MF of the related genes in the same pathways were showed in Figures 7D–F based on GO function analysis, respectively. Meanwhile, the KEGG pathway analysis was performed and the PPI network relationship were constructed about the related genes in the same pathways, then the results were separately showed in Figures 7B,C.

### GO Function and PPI Network Analysis for the Hub Genes

**FIGURE 8:**
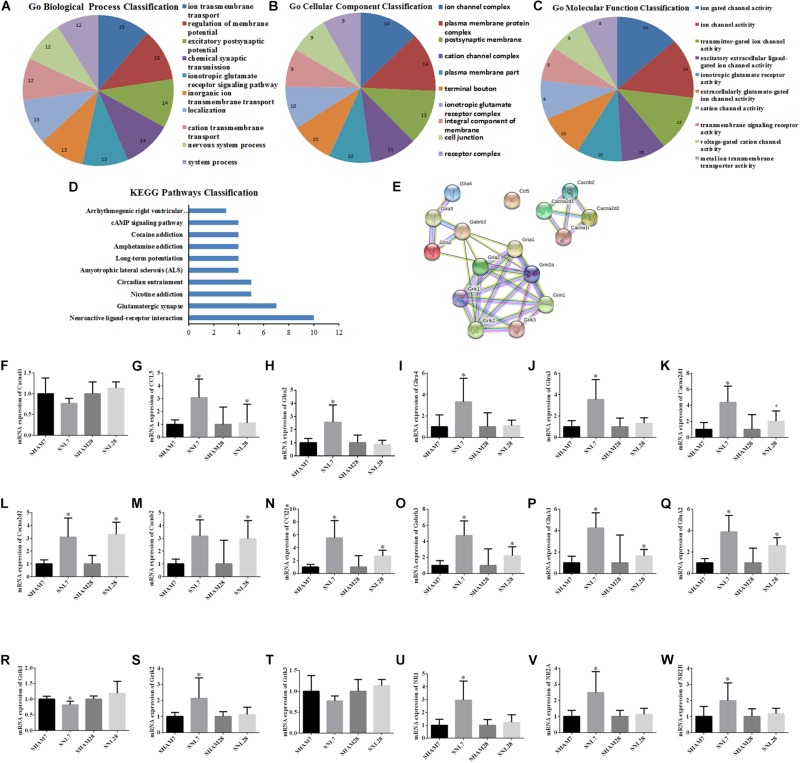
GO function and PPI network analysis of hub genes and PCR validation. **(A–C)** GO function analysis of the hub genes including biological process, cellular component and molecular function; **(D)** KEGG Pathways Classification of hub genes; **(E)** protein–protein interaction networks of hub genes of the conjunct pathways; **(F–W)** PCR validation for the expression of hub genes. ^∗^*P* < 0.05, compared with Sham-7d/28d group (SNL-7d group vs. Sham-7d group; SNL-28d group vs. Sham-28d group).

**FIGURE 9:**
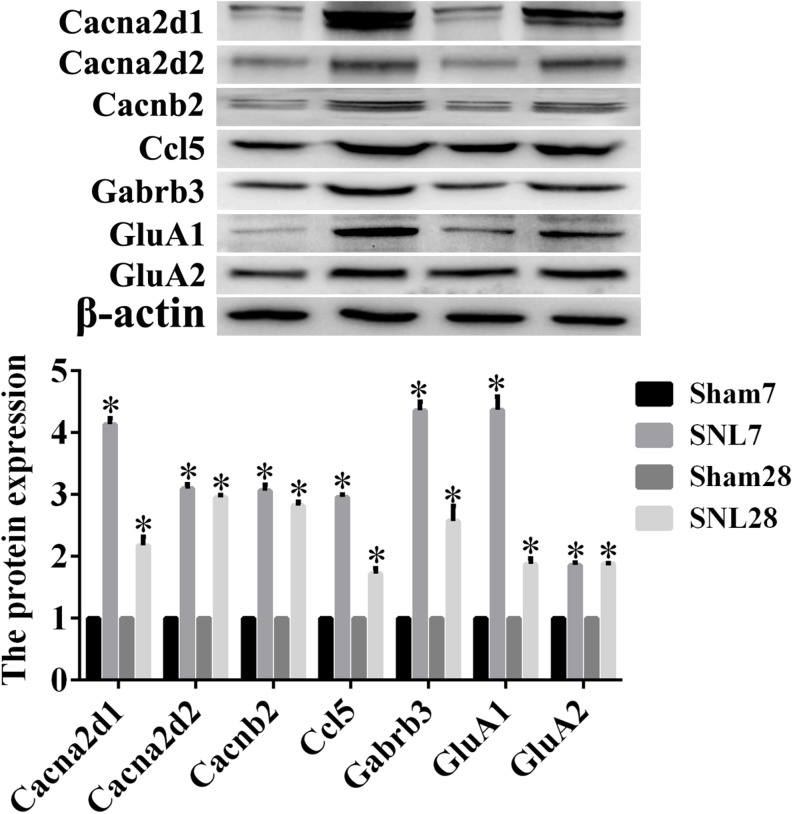
The protein expression detected by Western blot assay. Western blot assay was employed to measure the protein level, and this graph showed the protein expression including Ccl5, Cacna2d1, Cacna2d2, Cacnb2, Gabrb3, GluA1, and GluA2 in SNL-7/28d group and Sham-7/28d group, respectively. ^∗^*P* < 0.05, compared with Sham-7/28d group.

The candidate molecules were selected according to the bioinformatics prediction and literature report. Thereafter, these hub genes were analyzed by using bioinformatics methods and the mRNA expression were determined by qRT-PCR. GO function analysis of hub genes revealed that BP, CC, and MF mainly comprised of 119, 31 and 10 categories, and the top10 of these GO terms was showed in Figures 8A–C, respectively. Meanwhile, the results of KEGG pathway analysis and PPI network relationship of hub genes were showed in Figures 8D,E, respectively. Moreover, the results showed that the expression of CCL5, Cacna2d1, Cacna2d2, Cacnb2, Ccl21a, Gabrb3, GluA1, and GluA2 was significantly higher in SNL-7/28d group than that of in Sham-7/28d group (SNL-7d vs. Sham-7d; SNL-28d vs. Sham-28d; Figures 8G,K–Q, *P* < 0.05). And the level of Glra2, Glra4, Glra3, Grik1, Grik2, NR1, NR2A and NR2B was obviously increased in SNL-7d group compared to Sham-7d group (Figures 8H–J,R,S,U–W, *P* < 0.05), but which was no statistical difference between SNL-28d group compared to Sham-28d group (Figures 8H–J,R,S,U–W, *P* > 0.05). However, the mRNA expression of Cacna1i and Grik3 was no significant difference between SNL-7/28d group and Sham-7/28d group (SNL-7d vs. Sham-7d; SNL-28d vs. Sham-28d; Figures 8F,T, *P* > 0.05). Moreover, the protein level of Ccl5, Cacna2d1, Cacna2d2, Cacnb2, Gabrb3, GluA1, and GluA2 was obviously elevated in SNL-7/28d group compared to that of in Sham-7/28d group (SNL-7d vs. Sham-7d; SNL-28d vs. Sham-28d; Figure 9; *P* < 0.05).

## Discussion

NP is an intractable clinical problem characterized by complex symptoms, difficult treatments and poor outcomes (Haanpaa et al., 2011). Multiple causes jointly participated in the initiation of NP comprising of trauma, metabolic diseases, tumor invasion, infection, and neurotoxic chemicals (Cohen and Mao, 2014). To data, NP could not be completely cured derived from its complicated pathogenesis involving in excessive neurotransmitters, receptors, genes and modulatory pathways (Gonzalez-Ramirez et al., 2017). To investigate the underlying mechanism with respect to NP, the gene expression differentiation was detected at 7 and 28 days after operation through RNA-Seq. Thereafter, the DEGs responding to operative procedures were analyzed by using bioinformatics methods. The results showed that large volume of genes were significantly changed after surgery. To clearly understand the potential gene expression tendency, the top 2000 genes of DEGs were divided into four groups by using *k*-means clustering, and the result suggested synaptic signaling and neuropeptide signaling pathway were elicited after operation, which could effectively respond to nociceptive stimulus. Meanwhile, a hierarchical clustering tree and network were constructed to visualize the relationship among enriched GO terms. And the results revealed that enrichment network of DEGs mainly consists of wound healing, muscle structure development, neurotransmitter transmitting, cell-cell signaling and so on. The visualization graphics obtained from iDEP analysis tools displayed this biological process directly and visually. Briefly, the genes related wound healing and muscle structure development were upregulated and other parts linked to neurotransmitter transmitting and cell–cell signaling crosstalk were downregulated. We attempted to screen the key genes and signaling pathways by analyzing the DEGs at different point of time post operation. And the results would conduce to explore the role of these genes, and select certain key molecules in the process of repairing the tissues after surgery. Though we found numerous DEGs, the accurate mechanism still not be completely illuminated. Therefore, more information hidden behind gene sequence data need to be find out and then provided more value for treating SNL.

### Complicated Respondence of DEGs With Time After Surgery

Operative traumatic reaction results in complex physiological responses, and induces SNL. Our results displayed that 133 genes collectively involved in biological reactions of 7th and 28th day after surgery. BP analysis results showed that proton transmembrance transport, ATP metabolic process, and electron transport chain were mainly participated in this process. Meanwhile, the predication obtained from String database showed that Cox5a, Cox5b, Cox7c, and CYTB were involved in this BP simultaneously. XiYang et al. (2016) found that sodium channel voltage-gated beta 2 (SCN2B) played a crucial role in regulating synaptic plasticity associated with COX5A mRNA levels. Meanwhile, previous studies suggested that the loss of Cox5a/b contributed to mitochondrial dysfunction and thereby induced cell apoptosis (Cui et al., 2006). In this study, the results suggested that Cox subunits may exert an important function in promoting the transmission of pain messages. Moreover, the KEGG pathway analysis showed that MAPK family signaling cascades implicated in this procedures. Researches demonstrated that MAPK pathways played essential roles in anti-inflammation and tissue remodeling (Newton and Dixit, 2012), and inhibiting MAPK pathways effectively attenuated SNL in rats with chronic compression of the dorsal root ganglion (Qu et al., 2016). Therefore, the GO function analysis results for the conjunct DEGs at 7th and 28th day after surgery suggested that MAPK pathways and Cox subunits may play a pivotal role in regulating the nociceptive transmission. Whereas, the special mechanism still not be sufficiently uncovered due to its complexity. The dynamically regulated networks of DEGs with time needed to be further investigated in future.

### The Role of Hub Genes and Signaling Pathways in Rats With SNL

To clarify the potential mechanism and identify the target genes, GO function and PPI network analysis for the conjunct DEGs involved in the same pathways at 7th and 28th day after surgery were performed using String database. The results showed that retrograde endocannabinoid signaling and cardiac muscle contraction collectively participated in the procedure. Currently, researches showed that retrograde endocannabinoid signaling could mediate excitatory synaptic (Haj-Dahmane et al., 2018), and GABAergic synaptic transmission (Farkas et al., 2010). Meanwhile, retrograde endocannabinoid signaling regulated axonal growth and derived adult neurogenesis (Gao et al., 2010). So we speculated that retrograde endocannabinoid signaling maybe a core component in inducing SNL. Interestingly, the key genes of retrograde endocannabinoid signaling also comprised Cox5a and Cox5b. Therefore, we put forward to Cox5a and Cox5b maybe the key modulate units. Moreover, the hub genes were analyzed and their expression were determined. The results showed that the expression of CCL5, Cacna2d1, Cacna2d2, Cacnb2, Ccl21a, Gabrb3, GluA1, and GluA2 was significantly increased in SNL-7/28d group than that of in Sham-7/28d group. Interestingly, previous studies revealed that Cacna2d1 to 3, Ccl5, GluA1, and GluA2 were significantly implicated with the occurrence of pain (Bellessort et al., 2018; Capossela et al., 2018; Taylor et al., 2019), which were consistent with our results. Whereas, there has been virtually no reporting on dynamically studying the key molecular expression alteration. Similarly, the results suggested that the level of Glra2, Glra4, Glra3, Grik1, Grik2, NR1, NR2A, and NR2B was obviously increased in SNL group at 7 days following surgery, but which was no significant difference at 28 days. Related studies showed that NMDARs (including NR1, NR2A, and NR2B) may be a therapeutic target for preventing pain (Zhu et al., 2015; Gong et al., 2017), and our data also suggested that NMDARs may play a crucial role in regulating the pain sensation. Besides, the results showed that these genes expression was increased firstly and then remained unchanged. Therefore, this indicated these molecular may mainly play a role in early stage, and gradually return to normal with time. Collectively, this research preliminarily described the variation tendency of gene expression, while the special mechanism could not be clarified due to the complexity. Moreover, the KEGG pathway analysis documented that the hub genes enriched in the Glutamatergic synapse signaling. In addition, the BP analysis showed that the hub genes involved in synapse organization, excitatory postsynaptic potential, modulation of chemical synaptic transmission, and synaptic transmission. Nerve injury upregulated excitatory synaptic transmission in the dorsal horn, and then ultimately leaded to NP (Yu et al., 2019). The positive allosteric modulator (adenosine A1 receptor) selectively suppressed primary afferent synaptic transmission and thereby relieved syndromes in a NP model (Imlach et al., 2015). Furthermore, Kato E pointed out that antinociceptive action of PGB may involve in reducing spinal d-serine content and subsequent attenuating NMDA receptor-mediated synaptic transmission in the superficial dorsal horn (Kato et al., 2017). We found the possible key elements involved in the initiation and development of NP, but whether these genes were crucial still need to be further identify. Notwithstanding, this researches would provide new perspectives and strategies on deeply illuminating the underlying mechanism of NP.

## Data Availability Statement

The datasets generated for this study can be found in the NCBI Sequence Read Archive (SRA) database (SRA accession: PRJNA589837).

## Ethics Statement

All experimental procedures were approved by the Animal Care and Use Committee of Zhejiang University, and in accordance with guidelines for laboratory animal care and safety from NIH.

## Author Contributions

HY, X-YaL, and GC took part in the design of the experimental protocols. HY, Y-RC and X-YiL carried out experimental operation. PZ and Y-JW were in charge of the data analysis and drafting the manuscript. All authors read and approved the final manuscript.

## Conflict of Interest

The authors declare that the research was conducted in the absence of any commercial or financial relationships that could be construed as a potential conflict of interest.

## Footnotes

1 http://www.bioinformatics.babraham.ac.uk/projects/trim_galore/

2 https://github.com/trinityrnaseq/trinityrnaseq/wiki

3 http://bioinformatics.sdstate.edu/idep/

4 https://string-db.org/cgi/input.pl

5 http://bioinfogp.cnb.csic.es/tools/venny/index.html

## Supplementary Material

TABLE S1 Summarization for the previous RNA-seq work in pain areas.

DATA SHEET S1–S11 Raw data of analysis.
