# Supplementary material for: Temporal Changes of Spinal Transcriptomic Profiles in Mice With Spinal Nerve Ligation
Source: Front Neurosci. 2019 Dec 17;13:1357. doi: 10.3389/fnins.2019.01357 (PMC6928122; doi:10.3389/fnins.2019.01357)
Supplement: TABLE S1 — Summarization for the previous RNA-seq work in pain areas. [file Table_1.docx]

**Table. S1 Summarization for the previous RNA-Seq work in pain areas**

| **Technology** | **Species** | **Sample types** | **Disease** | **Doi** |
| --- | --- | --- | --- | --- |
| Whole exome sequencing | Human | Buccal samples | Complex regional pain syndrome | 10.1186/s13099-019-0311-z |
| RNA-sequencing | Rat | L4-6 dorsal root ganglia | Complex regional pain syndrome | 10.2147/JPR.S188758 |
| Whole exome sequencing | Human | Serum | Abdominal pain | 10.3760/cma.j.issn.0578-1426 |
| RNA-Seq | Rat | Forebrain cortex | Chronic pain | 10.3389/fnmol.2019.00005 |
| RNA-Seq | Human | synovia in the recessus suprapatellaris | knee pain | 10.3390/genes9070338 |
| RNA-Seq | Human/Mouse | Dorsal root ganglia | Chronic pain | 10.1097/j.pain.0000000000001217 |
| Whole exome sequencing | Mouse | Dorsal root ganglia | pain insensitivity disorder | 10.1093/brain/awx326 |
| RNA-Seq | Mouse | Dorsal root ganglia | Neuropathic pain | 10.1038/s41598-017-16664-z |
| Whole exome sequencing | Human | Blood | Paroxysmal extreme pain disorder | 10.1155/2016/9212369 |
| RNA-Seq | Mouse | Spinal cord | Chronic pain | 10.1016/j.celrep |
| Whole exome sequencing | Rat | Dorsal root ganglia | Chronic pain | 10.1126/scitranslmed.3007017 |
| RNA-Seq | Rat | Dorsal root ganglia | Neuropathic pain | 10.1186/1744-8069-10-22 |
| RNA-Seq | Rat | Dorsal root ganglia | Neuropathic pain | 10.1186/1744-8069-10-7 |
| RNA-seq | Rat | Dorsal root ganglion | Neuropathic pain | 10.1101/gr.101204.109 |
